# Bringing Together Evolution on Serpentine and Polyploidy: Spatiotemporal History of the Diploid-Tetraploid Complex of *Knautia arvensis* (Dipsacaceae)

**DOI:** 10.1371/journal.pone.0039988

**Published:** 2012-07-05

**Authors:** Filip Kolář, Tomáš Fér, Milan Štech, Pavel Trávníček, Eva Dušková, Peter Schönswetter, Jan Suda

**Affiliations:** 1 Department of Botany, Faculty of Science, Charles University in Prague, Prague, Czech Republic; 2 Institute of Botany, Academy of Sciences of the Czech Republic, Průhonice, Czech Republic; 3 Department of Botany, Faculty of Science, University of South Bohemia, České Budějovice, Czech Republic; 4 Institute of Botany, University of Innsbruck, Innsbruck, Austria; University of Lausanne, Switzerland

## Abstract

Polyploidization is one of the leading forces in the evolution of land plants, providing opportunities for instant speciation and rapid gain of evolutionary novelties. Highly selective conditions of serpentine environments act as an important evolutionary trigger that can be involved in various speciation processes. Whereas the significance of both edaphic speciation on serpentine and polyploidy is widely acknowledged in plant evolution, the links between polyploid evolution and serpentine differentiation have not yet been examined. To fill this gap, we investigated the evolutionary history of the perennial herb *Knautia arvensis* (Dipsacaceae), a diploid-tetraploid complex that exhibits an intriguing pattern of eco-geographic differentiation. Using plastid DNA sequencing and AFLP genotyping of 336 previously cytotyped individuals from 40 populations from central Europe, we unravelled the patterns of genetic variation among the cytotypes and the edaphic types. Diploids showed the highest levels of genetic differentiation, likely as a result of long term persistence of several lineages in ecologically distinct refugia and/or independent immigration. Recurrent polyploidization, recorded in one serpentine island, seems to have opened new possibilities for the local serpentine genotype. Unlike diploids, the serpentine tetraploids were able to escape from the serpentine refugium and spread further; this was also attributable to hybridization with the neighbouring non-serpentine tetraploid lineages. The spatiotemporal history of *K. arvensis* allows tracing the interplay of polyploid evolution and ecological divergence on serpentine, resulting in a complex evolutionary pattern. Isolated serpentine outcrops can act as evolutionary capacitors, preserving distinct karyological and genetic diversity. The serpentine lineages, however, may not represent evolutionary ‘dead-ends’ but rather dynamic systems with a potential to further influence the surrounding populations, e.g., via independent polyplodization and hybridization. The complex eco-geographical pattern together with the incidence of both primary and secondary diploid-tetraploid contact zones makes *K. arvensis* a unique system for addressing general questions of polyploid research.

## Introduction

Serpentine soils, characterized by specific chemical (i.e., low Ca/Mg ratio, high heavy metal content, low nutrient availability) and physical (e.g., drought) properties, strongly influence the plant life that grows on them [1], [2]. Although serpentines cover only 1% of dry land surface [3], they are nearly ubiquitous. The worldwide occurrence of serpentine-specific plant endemism highlights the global significance of serpentines in creating and preserving plant diversity. For example, more than 10% of the endemic Californian flora is restricted to serpentines, although serpentine soils make up less than 1% of the state’s surface [4].

From an evolutionary point of view, serpentine-rich areas represent ‘natural laboratories’, allowing researchers to address various evolutionary questions of general significance [1]. The unique features of serpentine soils can shape plant evolution in two main ways [5]–[7]. Firstly, they can act as a selective factor, picking tolerant genotypes out of mainly non-tolerant gene pools of potential colonizers. Such disruptive selection may result in ecotypic differentiation [8]–[10] and, provided that reproductive isolation is achieved, it may lead to sympatric or parapatric speciation of serpentine endemics on the border of serpentine area [5], [7], [11]. Secondly, the exclusion of many non-tolerant species from serpentine sites makes the localities a ‘light island’, where competitively weak but tolerant species can thrive. During dramatic environmental changes such as the climate fluctuations during the Holocene, non-serpentine populations may become regionally extinct due to massive vegetation shifts such as the postglacial reforestation. The surviving relict serpentine populations could then differentiate by means of allopatric speciation into separate taxa [12], [13]. Considering the island-like distribution of serpentine outcrops [4], [6], the spatially isolated populations of a serpentinophyte can ultimately give rise to several local endemics [14]. The evolutionary history becomes even more complicated if the serpentine populations come into secondary contact with their non-serpentine counterparts (e.g., after the progenitor’s re-invasion) and hybridize [15].

Serpentines may be viewed as an environmental trigger that can catalyze any evolutionary process [5]. Polyploidy (genome duplication), as a ubiquitous phenomenon in plants [16], [17], is generally acknowledged as a leading force in plant sympatric speciation [18]. Amongst other, polyploid taxa can have wider ecological amplitudes in comparison with their diploid counterparts, and this may result in distinct eco-geographic patterns [19]–[22]. Autopolyploids, i.e., polyploids with all sets of chromosomes derived from the same species, are particularly useful for studying ecological consequences of genome duplication because (i) di- and polyploid cytotypes are genetically very similar, and (ii) recurrent origins of autopolyploids may give rise to several lineages evolving under different selective pressures [23]–[25]. Despite the wide range of knowledge documented on the individual processes of serpentine and polyploid evolution, virtually no information is available on how these processes act in concert. Two scenarios, how serpentine differentiation interacts with polyploidy, can be invoked: (i) challenging abiotic conditions of serpentine habitats might support their colonization by more plastic polyploids, and (ii) low competitive environment of serpentine outcrops might enable relict survival of diploid lineages. To date, however, the relationships between evolution of serpentinophytes and karyological variation have been studied in a few diploid [26] or polyploid [27] plant groups and the results showed no clear patterns in the distribution of cytological variation and/or serpentine preferences.

The common European herb *Knautia arvensis* (Dipsacaceae) and its closest relatives constitute an intricate diploid-tetraploid complex exhibiting a distinct serpentine vs. non-serpentine habitat differentiation pattern in central Europe [19], [28], [29] and therefore provide an ideal system for investigations of the concerted action of genome duplication and a serpentine syndrome in plant evolution. Polyploidy, allopatric differentiation, and frequent homoploid hybridization are considered the major forces in the evolution of the complex; their interactive effects resulted in ambiguous species delimitation and fairly provisional taxonomic concepts [28], [30]. In contrast to frequent homoploid hybridization, strong reproductive barriers exist between 2× and 4× *Knautia* plants as indicated by the lack of triploid hybrids in sites with cytotype mixtures [31] and both tri- and tetraploid hybrids in artificial crossing experiments [28], [30], [32].

**Figure 1:**
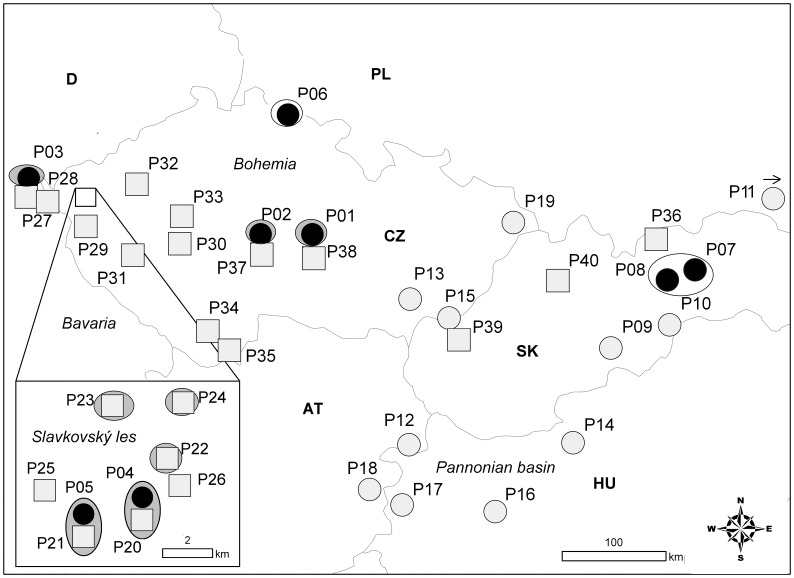
Ploidy level, genome size and habitat differentiation of the examined populations of *Knautia arvensis* agg. Light grey circles – diploids from ‘non-relict’ genome size group, black circles – diploids from ‘relict’ genome size group, squares – tetraploids, white ovals – relict limestone habitats (open pine forests or subalpine grasslands), grey ovals – relict serpentine pine forests; the remaining populations inhabit semiruderal grasslands (ploidy levels according to ref. 31). The map covers the region of eastern part of central Europe, the inset displays the situation in the diploid-tetraploid serpentine area in the Slavkovský les Mts.

There are two to three species of *K. arvensis* agg. in central Europe, which show a distinct pattern of geographic, karyological and edaphic differentiation (Fig. 1). In addition to the West Carpathian endemic tetraploid taxon *K. kitaibelii* (Schult.) Borbás, the widespread *K. arvensis* (L.) Coult. s.str. falls into two mostly parapatric cytotypes: diploids (2n = 2× = 20) occurring mainly in the southeastern part of central Europe, and tetraploids (2n = 4× = 40) occupying the northwestern half of the region. These two cytotypes are morphologically very similar and both prefer semiruderal mesophilous grasslands influenced by man [33]. In addition, several spatially isolated diploid populations of *K. arvensis* s.str. have been detected in markedly different habitats such as open pine forests on serpentine outcrops and subalpine grasslands in a glacial cirque [34]–[36] (Fig. 1). Open pine forests and subalpine communities of central Europe are regarded as classical examples of relict stands (i.e., supporting vegetation similar to that in the early Holocene [37]) that preserve significant plant diversity by providing an environment with low competitive pressure [13], [38], [39]. Moreover, similar relict habitats are preferred by *K. slovaca* Štěpánek, a diploid endemic taxon of central Slovakia with an unresolved taxonomic position, which was formerly not distinguished from *K. arvensis* s.str. [40] (Fig. 1). Interestingly, *K. arvensis* populations from relict stands and *K. slovaca* share identical genome size, significantly different from widespread semiruderal *K. arvensis* diploids [31]. For the sake of simplicity the two diploid groups with distinct genome size and habitat preferences will be termed ‘relict’ and ‘non-relict’ diploids hereafter. Finally, a serpentine tetraploid cytotype occurs in one serpentine area (the Slavkovský les Mts.; see inset in Fig. 1), forming both ploidy-uniform populations and diploid-tetraploid cytotype mixtures. Independent *in situ* autopolyploidization from local relict diploids has been suggested based on very similar morphology and ecological preferences [34], identical monoploid genome size, and co-occurrence of both cytotypes in several populations [31].

We employed two molecular markers that provide complementary information (AFLPs and plastid DNA sequences) to elucidate the evolutionary connection between evolution on serpentine and polyploidization in 40 populations of the *K. arvensis* agg. from Central Europe. This geographic restriction is justified by preliminary sequence and AFLP data (I. Rešetnik, P. Schönswetter & B. Frajman, unpubl.) suggesting that all the relict diploid central European populations of *K. arvensis* are genetically divergent from those elsewhere, e.g. on the Balkan Peninsula. Here, we addressed the following questions: (1) What are the genetic relationships among the species, cytotypes, genome size groups, and edaphic types within central Europe? (2) Is there any genetic differentiation at the diploid level? Do the two diploid groups with distinct genome sizes and divergent habitat preferences (i.e., relict and non-relict diploids) also represent separate genetic lineages? If so, is there any further genetic sub-structuring, e.g., according to geography and/or occupied habitat? (3) Did the serpentine tetraploids originate by recurrent (auto)polyploidization or by colonization of serpentine sites by non-serpentine tetraploids? (4) What are the relationships among serpentine and surrounding non-serpentine tetraploids? Is there indication of hybridization across the borders of serpentine areas?

## Materials and Methods

### Field Sampling

Plant materials were sampled from 2005 to 2008 in the Czech Republic, Slovakia, Hungary, Austria, Germany, and Ukraine. Because our study aimed at elucidating the evolutionary history of the complex in central Europe, with a particular attention to serpentine populations, the sampling scheme has been adapted to this purpose. Specifically, 34 populations of *K. arvensis* s.s., two populations of both *K. kitaibelii* and *K. slovaca*, and two populations of the introgressive hybrid of *K. arvensis* s.s. and *K. kitaibelii* (determined by morphology according to ref. [33]) were investigated. The resulting set of 40 populations covered the entire taxonomic, morphological and karyological diversity of *K. arvensis* agg. in central Europe. More intense sampling was performed in a serpentine ‘archipelago’ of the Slakovský les Mts. (western Bohemia), where large ecological and ploidy variation (including mixed-ploidy populations) was detected in our previous study [31]. Diploid and tetraploid subpopulations at two mixed-ploidy sites from this area (P04+ P20 and P05+ P21; see Table 1) were treated as separate populations in all analyses, considering strong inter-ploidy reproductive barriers [30]–[32]. At each locality information on the habitat type was gathered, accompanied by data from geological maps (scale 1∶25000; www.geology.cz) and vegetation surveys (e.g., ref. [41]); the status of serpentine sites has been also confirmed by soil analyses (R. Sudová et al., unpubl.). Leaves from approximately ten plants per population were collected and quickly desiccated in silica gel; to avoid collecting same genets, the distance between sampled individuals was at least 1 m. For each individual, flow cytometric results gained in our previous study [31] were available. The species under investigation is neither endangered nor protected and no specific permits were required to collect the plant samples at studied sites. Locality details, ploidy levels, genome size groups, and numbers of analyzed plants are summarized in Table 1. Vouchers have been deposited in the herbarium of the Faculty of Science, University of South Bohemia, České Budějovice (CBFS).

**Table 1:** Details on the 40 populations of *Knautia arvensis* agg. included in the study.

Code	Locality name a	Ploidylevel	Habitat b	Genome size group c	Taxon d	N	DW e	Nei’s gene diversity	FRAG f	% POLY g	cpDNA sequences h	Locality no. i
P01	CZ – Staré Ransko	2×	R-S	2×R	*K. arv.*	10	**0.61**	0.165	**93**	41.1	H (2)	71
P02	CZ – Borovsko	2×+	R-S	2×R	*K. arv.*	10	**0.56**	0.167	**91**	**44.2**	A (1), F (3)	263
P03	D – Woja	2×	R-S	2×R	*K. arv.*	10	**0.51**	0.137	87	34.9	A (2), B (1), G (1), I (1)	279
P04	CZ – Planý vrch (2×)	2×	R-S	2×R	*K. arv.*	10	0.31	0.141	81	36.4	D (3), L (1)	278
P05	CZ – Vlček (2×)	2×	R-S	2×R	*K. arv.*	4	**0.53**	0.158	83	28.7	–	277
P06	CZ – Krkonoše	2×	R-C	2×R	*K. arv.*	8	0.27	0.169	75	**45.0**	E (4)	72
P07	SK – Branisko	2×	R-L	2×R	*K. slov.*	10	0.29	0.143	76	38.8	A (3)	286
P08	SK – Lesnica	2×	R-L	2×R	*K. slov.*	8	0.38	**0.182**	83	41.9	–	284
P09	SK – Podrečany	2×	N	2×N	*K. arv.*	9	0.37	0.157	75	39.5	A (1)	58
P10	SK – Plešivec	2×	N	2×N	*K. arv.*	11	0.43	0.163	80	43.4	A (2)	61
P11	UA – Lviv	2×	N	2×N	*K. arv.*	5	0.29	0.147	68	29.5	–	70
P12	AT – Apetlon	2×	N	2×N	*K. arv.*	9	0.43	0.162	77	41.1	–	2
P13	CZ – Archlebov	2×	N	2×N	*K. arv.*	8	0.44	**0.175**	74	43.4	A (1)	31
P14	HU – Csobánka	2×	N	2×N	*K. arv.*	9	0.38	0.139	70	35.7	–	50
P15	CZ – Javorník	2×	N	2×N	*K. arv.*	9	0.37	0.173	78	44.2	–	19
P16	HU – Veszprém	2×	N	2×N	*K. arv.*	10	0.46	**0.202**	88	**57.4**	A (1), J (1), M (1)	48
P17	HU – Szombathely	2×	N	2×N	*K. arv.*	10	0.43	**0.198**	**92**	**52.7**	–	49
P18	AT – Bernstein	2×	N	2×N	*K. arv.*	10	0.41	0.135	73	35.7	A (1)	1
P19	CZ – Morávka	2×	N	2×N	*K. arv.*	5	0.43	0.166	72	33.3	–	46
P20	CZ – Planý vrch (4×)	4×	R-S	4×	*K. arv.*	10	0.43	0.121	86	33.3	A (2), D (2), K (1)	278
P21	CZ – Vlček (4×)	4×	R-S	4×	*K. arv.*	9	**0.47**	0.116	**89**	22.9	A (2), K (1)	277
P22	CZ – Pluhův bor	4×	R-S	4×	*K. arv.*	11	0.39	0.132	88	40.3	A (4), B (1)	259
P23	CZ – Křížky	4×	R-S	4×	*K. arv.*	10	0.30	0.111	81	31.8	A (2)	260
P24	CZ – Dominova skalka	4×	R-S	4×	*K. arv.*	9	0.22	0.118	70	31.0	A (3), B (1)	261
P25	CZ – Kladská	4×	N	4×	*K. arv.*	9	0.27	0.110	74	29.5	B (1)	257
P26	CZ – Mnichov	4×	N	4×	*K. arv.*	10	0.32	0.115	82	34.1	A (3)	258
P27	D – Döhlau	4×	N	4×	*K. arv.*	8	0.33	0.166	84	40.3	A (2), H (2)	242
P28	CZ – Libá	4×	N	4×	*K. arv.*	10	0.40	0.121	87	36.4	–	224
P29	CZ – Planá	4×	N	4×	*K. arv.*	10	0.33	0.157	87	41.9	B (2)	221
P30	CZ – Příbram	4×	N	4×	*K. arv.*	7	0.34	0.174	84	41.1	A (2), H (1), I (1)	217
P31	CZ – Přeštice	4×	N	4×	*K. arv.*	9	0.39	0.131	85	35.7	F (2)	215
P32	CZ – Blšany	4×	N	4×	*K. arv.*	10	0.33	0.137	77	36.4	F (2)	225
P33	CZ – Koněprusy	4×	N	4×	*K. arv.*	10	0.26	0.133	78	38.0	–	223
P34	CZ – Křemže	4×	N	4×	*K. arv.*	10	0.41	0.151	88	38.8	A (2)	144
P35	CZ – Benešov n. Černou	4×	N	4×	*K. arv.*	8	0.44	**0.202**	**90**	**50.4**	–	126
P36	SK – Relov	4×	N	4×	*K. arv.*	2	–	0.124	63	12.4	A (3)	256
P37	CZ – Bernartice	4×	N	4×	*K. arv.×kit.*	8	0.40	0.142	79	34.9	B (1)	216
P38	CZ – Ždírec n. Doubravou	4×	N	4×	*K. arv.×kit.*	8	0.36	0.127	80	31.8	A (1), B (1)	218
P39	SK – Pustá Ves	4×	N	4×	*K. kit.*	2	–	0.147	76	14.7	F (1)	281
P40	SK – Sklabiňa	4×	N	4×	*K. kit.*	1	–	–	51	–	C (1)	283

a AT – Austria; CZ – Czech Republic; D – Germany; HU – Hungary; SK – Slovak Republic; UA – Ukraine.

b R – relict habitat, i.e., serpentine (R-S) or limestone (R-L) outcrops or a subalpine glacial cirque (R-C); N – non-relict habitat (mostly semi-ruderal mesophilous grassland).

c 2×R – relict diploid genome size group; 2×N – non-relict diploid genome size group; 4× – tetraploid genome size group according to ref. 31.

d *K. arv.* – *Knautia arvensis* s.s.; *K. kit.* – *Knautia kitaibelii*; *K. arv.*×*kit.* – *Knautia arvensis* × *K. kitaibelii*; *K. slov.* – *Knautia slovaca.*

e DW  =  weighted rarity index (only for populations with more than three individuals).

f number of fragments.

g percentage of fragments exhibiting intrapopulational polymorphism.

h list of different cpDNA haplotypes found in the population (numbers of sequenced individuals possessing the particular haplotype in brackets); for details see Fig. 4.

i Locality number in ref. 31 where details on geographic location of the localities as well as the results of flow cytometric analyses are provided.

+ a single triploid individual detected within population P02 was included in the AFLP analysis.

In each case, the five populations with the highest values of DW, Nei’s diversity, FRAG, and % POLY are highlighted in bold.

### AFLP Amplification and Scoring

Total genomic DNA was extracted using the Invisorb Spin Plant Mini Kit (Invitek) following the manufacturer’s instructions. In total, 336 individuals from 40 populations were analyzed for AFLPs using the AFLP Core Reagent Kit I (Invitrogen) and AFLP Pre-Amp Primer Mix I (Invitrogen). Restriction, ligation and pre-amplification followed Rejzková et al. [42], but with the restriction phase extended to five hours. Selective amplification was performed using 2.3 µL of 10 times diluted pre-amplification product as a template, 1 µL of 10× buffer for Ampli Taq Gold (Applied Biosystems), 0.2 mM dNTPs (Fermentas), 0.05 µM of EcoRI-selective fluorescence-labelled primer (Applied Biosystems), 0.25 µM of MseI-selective primer (Applied Biosystems), 0.5 U of Ampli Taq Gold (Applied Biosystems), 0.5 µL of 1.25 mM MgCl_2_ (Applied Biosystems) and 4.7 µL of ddH_2_O (total volume 9.8 µL). Three primer combinations were used for selective amplification: EcoRI-ACA (6-FAM labelled) + MseI-CTG, EcoRI-ACC (NED labelled) + MseI-CTC, and EcoRI-ACG (HEX labelled) + MseI-CTA. The reaction was placed in a Mastercycler ep gradient S thermal cycler (Eppendorf). Reaction conditions were an initial step of 2 min at 94°C, 30 s at 65°C and 2 min at 72°C, followed by eight cycles of 1 s at 94°C, 30 s at 64°C (reduced by 1°C per cycle), 2 min at 72°C, followed by 23 cycles of 1 s at 94°C, 30 s at 56°C, 2 min at 72°C, with a final extension time of 30 min at 60°C. For each sample, 1 µL of each 6-FAM-, NED- and HEX-labelled selective PCR product was pooled and precipitated using an ethanol/sodium acetate precipitation. The precipitate was resuspended in 10 µL deionized formamide and combined with 0.25 µL of GeneScan-ROX-500 size standard (Applied Biosystems). Fragments were resolved on a 3100 Avant Genetic Analyzer and scored with GeneMarker v 1.8 (www.SoftGenetics.com). Thirty-nine samples (12% of all samples) were re-analyzed by repeating the whole AFLP procedure from the extracted DNA onward in order to test reproducibility of the data by estimating the average proportion of correctly replicated bands [43]. Only bands in the range of 100–500 bp, which could be scored unambiguously, were included; those found by comparing replicate runs not to be reproducible were excluded from the analyses. The resulting presence/absence matrix was used in subsequent analyses.

### Plastid DNA Sequencing

Plastid DNA haplotype variation was assessed to complement the information given by the mainly nuclear AFLPs. The *pet*N(*ycf6*)*–psb*M region was sequenced for 77 accessions representing all the groups indicated by the AFLP analysis (see Table 1). More thorough haplotype sampling was performed in populations from the Slavkovský les serpentine area (i.e., a region with potentially recurrent polyploidization). PCR amplification with the primers *ycf6*F and *psb*MR of Shaw et al. [44] was carried out in a volume of 20 µl reaction using 5 ng of template DNA, 2 µl of 10× reaction buffer (Sigma), 0.4 µl of 10 mM dNTP mix (Fermentas), 6.25 pmol of each primer and 0.5 U of Jump Start REDTaq DNA Polymerase (Sigma) on a Mastercycler ep gradient S thermal cycler (Eppendorf) with initial denaturation at 94°C for 2 min, 35 cycles of 1 min denaturation at 94°C, 1 min annealing at 55°C and 2 min extension at 72°C, followed by 10 min final extension at 72°C. Amplification products were subsequently purified using the JetQuick PCR Purification Kit (Genomed). Sequencing reactions were performed using BigDye Terminator v3.1 Cycle Sequencing Kit (Applied Biosystems) according to the manufacturer’s instructions using the primers cited above. Purification of sequencing reactions was carried out using an ethanol/sodium acetate precipitation. Products were run on an ABI 3130 Genetic Analyzer (Applied Biosystems).

### AFLP Data Analyses

Nei’s gene diversity [45] (termed ‘genetic diversity’ in the following), an estimator of local genetic diversity that can be applied regardless of the ploidy level [46], was computed for each population with the R-script AFLPdat [47]. The same tool was used for the calculation of a rarity index by computing ‘frequency-down-weighted marker values’ per population (DW) [48]. Only populations with a sample size of more than three individuals were included in the computations. The DW is higher in populations or groups that harbour a high number of rare markers [49]. A two-tailed t-test (calculated using Statistica 8.0) was used for testing the differences in the DW and genetic diversity among particular groups defined by ploidy level and/or genome size.

The genetic structure was inferred using three independent approaches. (1) A non-model-based approach, nonhierarchical K-means clustering [50], was chosen because of the presence of two ploidy levels, and performed using a script of Arrigo et al. [51] in R. This approach has recently been successfully applied in the analysis of genetic structure of the AFLP dataset in polyploid complexes [51], [52]. We performed 50,000 independent runs (i.e., starting from random points) for each assumed value of K clusters ranging from 2 to 10. The first run yielding a positive value for the second derivative of the inter-cluster inertia was considered [52]. (2) In the model-based Bayesian clustering approach implemented in structure version 2.2 [53], [54], the number of clusters was estimated using 10^6^ iterations, with a burn-in period of 10^5^ iterations under an admixture model with recessive alleles. The number of clusters (K) was used as a prior value; ten replicates for each K were analyzed from K = 1 to K = 10. All analyses using structure were carried out at the Bioportal of the University of Oslo (www.bioportal.uio.no). To determine the most likely number of clusters we followed the approach of Evanno et al. [55] implemented in Structure-sum-2009 [47]. After the optimal grouping was determined, each group was analyzed separately under the same settings used for the main analysis. (3) K-means and structure clustering results were independently displayed on a principal coordinate analysis (PCoA) computed with the R package ADE-4 [56] based on a Jaccard distance matrix of the AFLP data. Finally, congruence of the two different clustering techniques was compared and tested using a contingency table (calculated in Statistica 8.0) and displayed on a map using ArcGIS 9.3 (ESRI).

The partitioning of genetic variation among the populations, species, cytotypes, and genome size groups was quantified using analyses of molecular variance (AMOVA). AMOVAs were conducted in Arlequin 3.11 [57]. For nested AMOVAs, the populations were divided into: (i) three species (*K. arvensis* s.str., *K. kitaibelii*, and *K. slovaca*); (ii) two ploidy levels (2×, 4×); and (iii) three main groups according to their ploidy level and monoploid genome size that also well correlated with the geographic distribution and habitat preferences (i.e., non-relict diploids, relict diploids, and tetraploids). This approach allowed us to assess the structuring of genetic variation according to both (i) traditional taxonomic concepts, and (ii) the patterns of eco- and cyto-geographical variation, irrespective of taxonomic assignments. In addition, separate AMOVAs were conducted for the mixed-ploidy area in the Slavkovský les Mts. in order to examine the level of differentiation among the diploid and putatively locally originated tetraploid cytotypes.

### Plastid DNA Data Analyses

Plastid DNA sequences were edited using Finch TV (Geospiza) and aligned in the MAFFT 6 online application using the default mode [58]. Haplotype networks were constructed using TCS version 1.21 [59], treating gaps as a fifth character state. For this purpose, insertions/deletions longer than 1-bp were treated as single-step events. The sequences together with voucher numbers are available at GenBank (accession no. HM597685-HM597697 for haplotypes A-M).

## Results

### AFLP Data

The three AFLP primer combinations yielded 129 clear polymorphic fragments (for primary data matrix see Table S2). Based on 39 replicates, the reproducibility of the dataset was 95%. All 336 individuals had different AFLP phenotypes. Genetic diversity (Table 1) varied approximately two-fold, from 0.110 in population P25 (non-relict 4×) to 0.202 in populations P35 (non-relict 4×) and P16 (non-relict 2×). The level of genetic diversity was significantly higher in the diploid than in the tetraploid populations (two-tailed t-test, df = 37, t = 3.65, p<0.001, mean values of 0.162 and 0.137 for 2× and 4×, respectively). The rarity index (DW; Table 1) varied by a factor of three, from 0.22 in population P24 (relict 4×) to 0.61 in population P01 (relict 2×). The DW values of the diploid populations were significantly higher than those of the tetraploids (df = 35, t = 2.27, p = 0.030, mean DW of 0.42 and 0.36 for 2× and 4×, respectively). Interestingly, the highest DWs corresponded to four diploid populations from relict serpentine stands (P01, P02, P03, and P05; see Table 1); this was also reflected in the significantly higher DW values of the serpentine diploids (df = 35, t = 3.89, p<0.001). Notwithstanding, the group of relict populations as a whole did not have significantly different DW values (df = 35, t = 0.99, p = 0.324).

**Figure 2:**
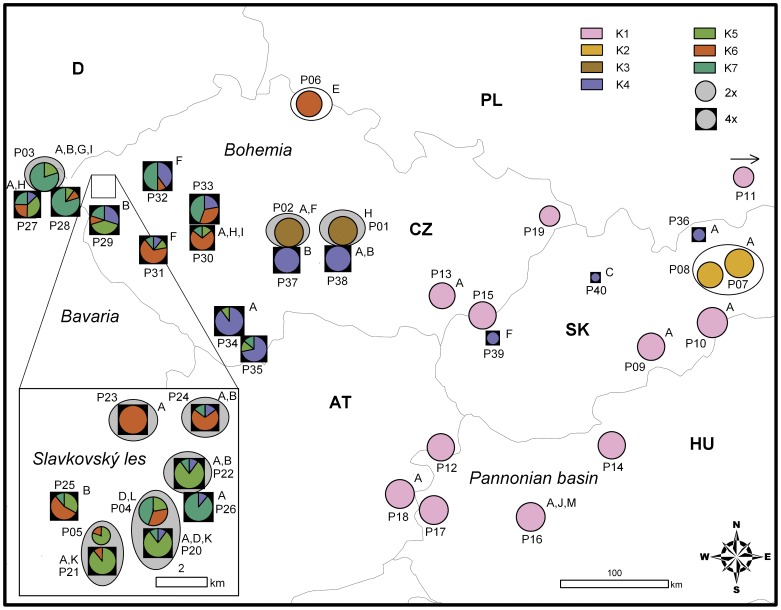
Phylogeographical grouping of 40 analyzed populations of *Knautia arvensis* agg. in central Europe. Grouping is according to the nonhierarchical K-means clustering of AFLP phenotypes. Pie charts represent the proportion of individuals belonging to each of the seven detected groups (K1–K7). The size of the pie chart reflects the sample size. The inset displays the situation in the Slavkovský les serpentine area. White ovals denote populations from relict limestone habitats (open pine forests or subalpine grasslands), grey ovals populations from relict serpentine pine forests. Note the presence of several relict diploid populations in the western part of the area (P03, P04, and P05) with the genetic composition highly similar to the surrounding tetraploids. The distribution of chloroplast haplotypes is indicated (A–M).

Nonhierarchical K-means clustering revealed an optimal separation of the dataset into seven groups (the second derivative of the inter cluster inertia was 1.99; Figure S1), mostly reflecting the ploidy level, genome size, and habitat differentiation. Separate clusters were formed by the (i) non-relict diploids (P09–P19), (ii) relict limestone diploids (P07 and P08, corresponding to *K. slovaca*), and (iii) eastern relict serpentine diploids (P01 and P02; see Fig. 2). The remaining western relict serpentine (P03–P05) and subalpine (P06) diploid populations were included in three clusters, which also contained tetraploid *K. arvensis* s.str. (clusters K5, K6, and K7). In addition, one exclusively tetraploid cluster (K4), formed by *K. arvensis* s.str. and *K. kitaibelii* populations, was recognized (Fig. 2). structure analysis of the entire data set revealed two main groups comprising (i) non-relict diploids, and (ii) relict diploids + all tetraploids (the highest, 0.99, similarity among runs and the highest delta K; Figures S2A and S2D). Separate structure analyses, run for each main group (excluding the two populations P07 and P08 that were highly admixed in the previous structure analysis of the entire dataset, Figure S3A), revealed no clear substructure within the non-relict diploids (a decreasing pattern of likelihood together with similarity coefficients below 0.36; Figures S2B and S2E), while the second main group was further divided into seven sub-groups (high, 0.97, similarity among runs and the highest delta K; Figures S2C and S2F). The structure groups (Figure S4) were congruent with the K-means clusters (chi-square = 924, df = 54, p<0.0001; for details see Table 2). High levels of congruence were achieved at the diploid level; the entirely diploid clusters were fully congruent and only four diploid individuals were assigned to a different structure vs. K-means group in the remaining clusters. Several tetraploid individuals were assigned to different clusters in K-means vs. structure clustering, what probably reflects generally lower genetic distinctness at the tetraploid level (as was also illustrated by higher genetic admixture of tetraploids, Figure S3B).

**Table 2:** Contingency table comparing the clustering results obtained by nonhierarchical K-means and structure analyses (numbers of individuals are presented in each field).

	S1	S2	S3	S4	S5	S6	S7	S8	S9	NA
K1	95									
K2		**18**								
K3			**17**							
K4				*31*	*1*	*1*	*6*		*11*	*6*
K5					***45***	*1*				*1*
K6					***3***	*21*	***12***	**8**	***6***	*4*
K7					***44***	*1*			***3***	*1*

Different font styles denote cytotypes with distinct monoploid genome size in the particular field (regular  =  non-relict diploids only, bold  =  relict diploids only, italics  =  tetraploids only, bold italics  =  relict diploids and tetraploids).

**Figure 3:**
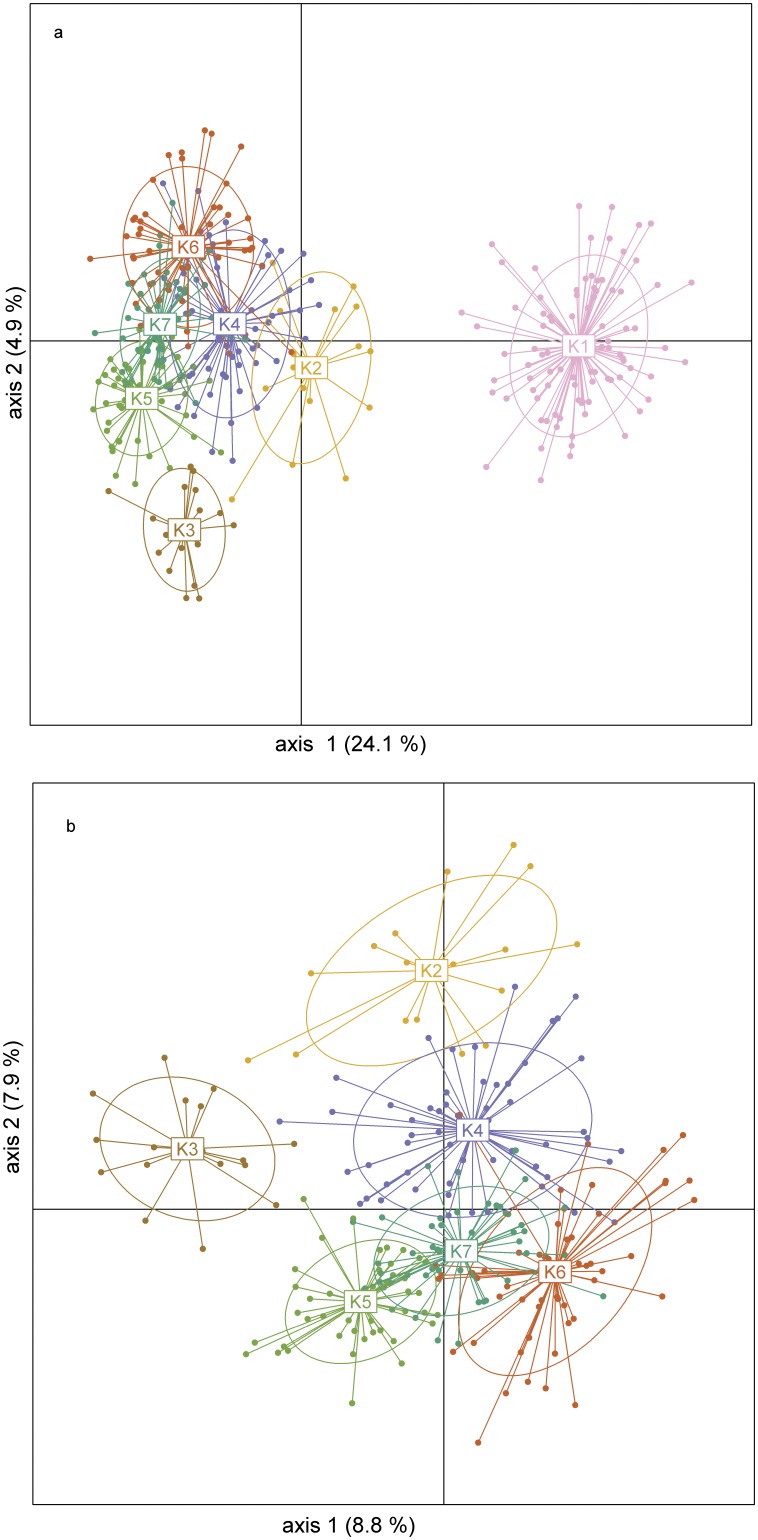
Principal coordinate analysis based on Jaccard similarity among AFLP multilocus phenotypes of *Knautia arvensis* agg. (a) entire data set; (b) excluding the most divergent group K1 (i.e., non-relict diploids). The different colours represent the groups identified by nonhierarchical K-means clustering (same as in Fig. 2). The centroid of each group and its connection with other points are displayed as well as an ellipse reflecting the variance of the group and the covariance on the axes.

The seven K-means clusters were also visible on the PCoA plot (Fig. 3a). The first axis (explaining 24.1% of the total variation) corresponds to the main split in the dataset, i.e., the separation of non-relict diploids (cluster K1) from the remaining samples (all tetraploids + relict diploids). Within the 4×+relict 2 group, the eastern serpentine populations (P01 and P02; cluster K3) and *K. slovaca* (P07 and P08; cluster K2) are well separated from the remaining clusters (Fig. 3b). Results of the structure clustering are displayed in Figures S5A and S5B.

AMOVA analyses (Table 3) attributed 37% of the overall genetic variation to the among-population component. In the nested AMOVAs, the variation between the two cytotypes accounted for 18.9% of the overall variation; conversely, species-based grouping explained only 4% of the variation. The highest values of among-population differentiation were found within the relict diploid group (30.5%), whereas the non-relict diploid populations were the least differentiated (14.3%). Interestingly, separate analysis of the mixed-ploidy area of the Slavkovský les Mts. yielded a fairly high (22.9%) inter-population variation while the differentiation between the local 2× and 4× cytotypes was negligible (0.7%).

**Table 3:** Analyses of molecular variance (AMOVA) of AFLP phenotypes of *Knautia arvensis* agg. grouped according to traditionally recognized species, ploidy levels, and cytotypes with distinct monoploid genome size values (according to ref. 31).

	d.f.	% of variation	Fst^a^
**A. Complete dataset**			
Among all populations	38	37.1	0.371
* Within populations*	*296*	*62.9*	
**Species grouping**			
Among species*	2	4.0	0.396
* Among populations within species*	*34*	*35.6*	
* Within populations*	*282*	*60.4*	
**Ploidy level grouping**			
Among all 2× vs. 4×	1	18.9	0.429
* Among populations within groups*	*37*	*24.0*	
* Within populations*	*296*	*57.1*	
**Genome size grouping**			
Among relict 2× vs. non-relict 2× vs. 4×	2	27.5	0.434
* Among populations within groups*	*36*	*15.9*	
* Within populations*	*296*	*56.6*	
Among populations of relict 2×	7	30.5	0.305
* Within populations*	*62*	*69.5*	
Among populations of non-relict 2×	10	14.3	0.143
* Within populations*	*84*	*85.7*	
Among populations of 4×	21	24.8	0.248
* Within populations*	*157*	*75.2*	
**B. Only Slavkovský les area**			
Among all populations in Slavkovský les	8	22.9	0.229
* Within populations*	*73*	*77.1*	
Among 2× vs. 4× in Slavkovský les	1	0.7	0.233
* Among populations within groups*	*7*	*22.6*	
* Within populations*	*73*	*76.7*	

a all p-values <0.001.

The two populations of an introgressive hybrid between *K. arvensis* and *K. kitaibelii* (P37, P38) were omitted from this analysis.

### Plastid DNA Data

**Figure 4:**
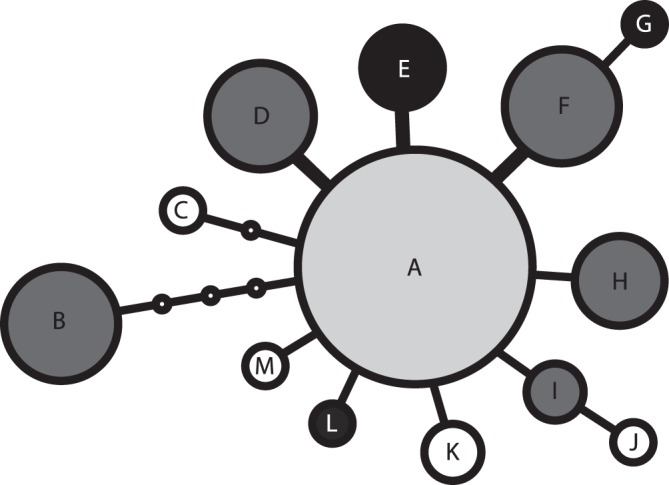
Network of 13 plastid DNA haplotypes found within 77 examined individuals of *Knautia arvensis* agg. The size of the circles is proportional to the number of individuals, while their shading indicates the ploidy level and monoploid genome size of the samples (black – relict 2× only, dark grey – relict 2×+4×, light grey – all 2×+4×, white – unique for a single non-relict 2× – haplotypes J and M – or 4× – haplotypes C and K – population). The double line indicates an insertion-deletion. For more detailed information, see Table 1.

Sixteen variable positions (including three coded indels) out of 497 aligned positions were detected. In total, 13 haplotypes were identified within the 77 sequences (Table 1). Half of the accessions belonged to the widespread haplotype A (Fig. 4), regardless of ploidy level, genome size or habitat preference. Globally, AFLP and plastid DNA data sets were not congruent (chi-square = 79.5, df = 74, p = 0.26; e.g., individuals from all AFLP groups possessed the single central haplotype A, for details see Table S1). Despite this, some interesting insights can be gained from the data. First, derived haplotypes of non-relict diploid populations (cluster K1) were not found in other populations; on the other hand, the relict diploids often shared haplotype with tetraploids (haplotypes B, D, F, H, and I; Fig. 4). Second, the isolated subalpine diploid population P06 from the cluster K6 is exclusively characterized by a 12-bp insertion (haplotype E). Finally, the haplotype D is exclusively shared by diploid and tetraploid individuals from the same mixed-ploidy serpentine population Planý vrch (P04 and P20) from the Slavkovský les Mts. (see Fig. 2 for details on haplotype distribution).

## Discussion

In this study, we took advantage of the ‘full-factorial’ pattern of ploidy variation (diploid vs. tetraploid cytotypes) and edaphic specialization (serpentine vs. non-serpentine populations) in *K. arvensis* agg. from central Europe in order to gain new insight into the evolutionary history of this polyploid plant system and, in particular, to assess how polyploid evolution can be connected with serpentine differentiation. Because of the incongruence between the traditional species delimitation and the inferred genetic structure we will discuss the evolutionary history of the central European populations of *K. arvensis* agg. regardless of their taxonomic assignment.

### Differentiation at the Diploid Level

The most pronounced genetic differences within the central European *K. arvensis* agg. were observed at the diploid level. Specifically, the non-relict diploid populations from the Pannonian basin and the Polonian lowlands (P09–P19; cluster K1) formed the most distinct group in the AFLP dataset (Fig. 3a). Moreover, these non-relict diploids also clearly differed in the size of their monoploid genome, i.e., the Cx-value [31]. Variation in genome size is often regarded as an indication of cryptic differentiation or incipient speciation [60]–[63]. The non-relict diploids can thus be regarded as a very distinct lineage within the central European *K. arvensis* agg.

The remaining diploids (collectively called relict diploids) differ from their non-relict counterparts by smaller genome size [31] and habitat preferences (they mostly grow in open relict pine forests with specific edaphic conditions whereas non-relict diploids grow in anthropogenic semiruderal grasslands). The AFLP markers revealed two distinct genetic clusters within the relict diploids, representing two geographically and ecologically well-characterized lineages (Fig. 3b). One lineage inhabits pine forests on limestone in central Slovakia (cluster K2, corresponding to *K. slovaca*) while the other lineage grows in open pine forests on isolated serpentine outcrops in central Bohemia (cluster K3; Fig. 2). The remaining relict diploid populations (from serpentine outcrops in western Bohemia and a subalpine glacial cirque in eastern Bohemia) contain individuals from three fairly close clusters (K5, K6, and K7; Fig. 3b), all of them containing also tetraploid plants. Furthermore, the relict diploids also exhibited the highest levels of inter-population genetic differentiation (above 30%; Table 3) what is also in line with the high number of identified groups. Collectively, this marked genetic differentiation together with specific habitat requirements may reflect long-term persistence in isolated open island habitats serving as refugia during Holocene reforestation. For the serpentine populations, long-term persistence is further supported by the accumulation of rare AFLP fragments (significantly higher DW values; Table 1). In addition, despite generally low congruence among AFLP and plastid DNA data (resulting from low discriminative power of the cpDNA data and probably also reflecting the effects of ancestral polymorphism, hybridization and/or recurrent polyploidization), serpentine diploid populations are distinct by their high incidence of rare plastid DNA haplotypes (six out of twelve rare haplotypes; Table 1). A high frequency of rare genetic markers is generally acknowledged as strong evidence for the relict status [48], [49], [64]. The origin of these relict diploid lineages seems strongly connected to serpentine habitats and is discussed in the section ‘Joining edaphic differentiation and polyploid evolution’. The non-exclusive hypothesis of independent immigration from other parts the range such as the Balkan Peninsula (i.e. diversity hotspot of the whole genus, see ref. [65]) is discouraged by phylogenetic data documenting an isolated position of the central European relict diploids among European diploid *Knautia* (I. Rešetnik, P. Schönswetter & B. Frajman unpubl.).

### Recurrent Polyploidization

Recurrent origin is now widely recognized as a frequent component of polyploid evolution that is responsible for the marked diversity of many polyploid complexes [16], [66]. Independently formed polyploid lineages can exhibit striking differences in morphology, ecology or genetic profiles, even if originating from the same ancestral source [67], [68]. In addition, distinct lineages can meet and hybridize, which further increases variation at the polyploid level [24].

The serpentine ‘archipelago’ in the Slavkovský les Mts., unlike any other central European relict locality, harbours a tetraploid *Knautia* cytotype. Here, we argue that the serpentine tetraploids were formed independently from their non-serpentine counterparts by independent autopolyploidization from a local diploid cytotype. Close evolutionary relationships between the local serpentine di- and tetraploids have previously been suggested on the basis of phenotypic similarities and habitat preferences [34], as well as cytogeographical patterns and identical monoploid genome size values [31]. Molecular data further support the hypothesis of local auto-polyploid origin of the serpentine tetraploids. Firstly, the diploid populations from the Slavkovský les Mts. grouped together with the surrounding tetraploids (see Fig. 2). Secondly, the AMOVA analysis revealed low differentiation between the co-occurring di- and tetraploids explaining only 0.7% of the total genetic variation in the Slavkovský les Mts. (Table 3). Finally, several di- and tetraploid individuals from the population Planý vrch (P04 and P20) share the same unique 6 bp insertion in their plastid DNA (haplotype D; see Table 1). The alternative hypothesis of strong introgression of the tetraploid genotype into the diploids can be ruled out due to the virtual lack of triploid hybrids [31]. Unidirectional introgression of 2× genotypes into established tetraploids via unreduced gametes alone cannot sufficiently explain such a high genetic similarity between both cytotypes. First, strong inter-ploidy reproductive barriers were indicated by several crossing experiments [28], [30], [32]. Second, even if the breeding barriers were overcome, vast amounts of viable unreduced gametes would be necessary for dissolving the original 4× genetic pool, which contrasts with the low frequency of unreduced gametes formation in general [69], [70], and in the *K. arvensis* agg. in particular [31]. Finally, there is no indication of across-ploidy genetic admixture in the other contact zone between the tetraploids and non-relict diploids in the Pannonian basin. To sum up, all lines of evidence such as genetics, cytology, morphology, and ecology point to at least one independent autopolyploidization event, which took place *in situ* in the Slavkovský les Mts., leading to an independent origin of serpentine tetraploids from local relict diploids.

The *K. arvensis* agg. exhibits two strikingly different types of contact zones between cytotypes in central Europe. The ploidy mixtures in the Slavkovský les Mts. arose as a result of *in situ* (auto)polyploidization (i.e., they are composed of almost identical genotypes) and thus fit well into the concept of a primary contact zone [71]. In contrast, ploidy-heterogeneous stands on the borders of other serpentine localities and, in particular, the diffuse contact zone among tetraploids and non-relict diploids in the Pannonian basin [31] represent zones of 2×/4× secondary contact where two distinct gene pools meet (see Fig. 2 and Fig. 3a). There are only a few other plant groups, including *Dianthus* [72], [73] and *Melampodium* [74], for which both primary and secondary contacts have been suggested, but these have never been confirmed by molecular markers. According to our knowledge, *K. arvensis* agg. thus represents the first polyploid system for which the incidence of both established primary and secondary contact zones has been supported by molecular evidence.

**Figure 5:**
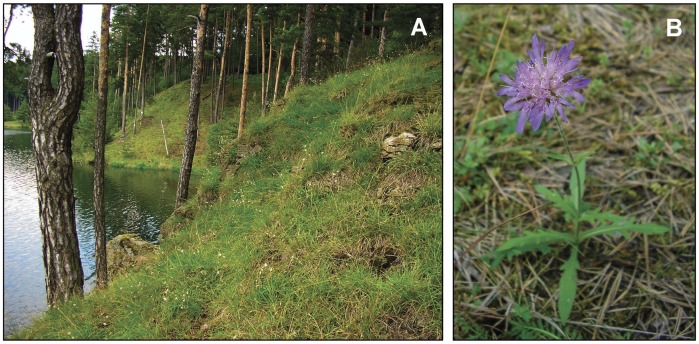
Serpentine outcrop covered by open pine forest near Borovsko, central Czech Republic (A.) This locality probably served as a Holocene refugium for several rare plant taxa. Morphologically distinct ‘relict diploid’ cytotype of *Knautia arvensis* (B, population P02 in this study) also occurs at this site.

### Joining Edaphic Differentiation and Polyploid Evolution

Serpentines can shape plant evolution either by the selection of tolerant genotypes from the colonizing populations or by providing refugia in island-like serpentine outcrops [6], [7]. In the latter case, vegetation shifts caused by climatic changes could cause local extirpation of the non-serpentine populations, while the subsequently isolated populations on serpentine may further evolve by means of allopatric differentiation and local adaptation into new taxa (i.e., the so-called ‘depleted species’ evolutionary scenario; [4]). The highly differentiated relict diploid populations of *K. arvensis* might fit into this model. Diploid ancestors may have been present in ice-free central Europe during the late Pleistocene as suggested by *Knautia* pollen records from the Allerød interstadial [75], [76]. Subsequently, the heliophilous plants were restricted to serpentine, limestone or subalpine refugia by the expanding forest vegetation (see the example of relict *Knautia* serpentine habitat in Fig. 5). As a consequence of spatial isolation and population size fluctuations, mechanisms of allopatric differentiation could have taken place, ultimately leading to the genetic and morphological differentiation currently observed among the relict diploid populations (see Fig. 2; cf. [34], [36]). Similar scenarios of speciation in isolated serpentine refugia were also suggested for several central European serpentine endemics – e.g., *Cerastium alsinifolium* [13], *Minuartia smejkalii* [77], and *Potentilla crantzii* subsp. *serpentini* [39]. Irrespective of the relative importance of allopatry vs. potential independent immigration, the highly differentiated diploid lineages within the *K. arvensis* agg. illustrate the significance of Holocene edaphic refugia for preserving rare and distinct genetic diversity.

Regarding the other *Knautia* lineages, i.e., tetraploids and non-relict diploids, it seems plausible that they immigrated into central Europe later as a result of human-induced landscape changes, such as deforestation, grazing, and meadow agriculture [34], [36]. This hypothesis corresponds well with the current semi-ruderal habitat preferences of both lineages [33]. Further details on the relationships and evolutionary history of these lineages, however, cannot be inferred without more intensive sampling in other parts of the range of *K. arvensis* agg. A similar scenario of range contraction into serpentine refugia, followed by human-enhanced re-colonization by different genotypes, has been suggested for Scandinavian populations of *Silene dioica* [78].

In addition to the above-discussed ‘depleted species-recolonization’ scenario, the serpentine *Knautia* populations underwent independent polyploid evolution – a process not yet recorded in the evolution of any other serpentine relict. Moreover, it seems that the genome duplication opened new possibilities for the serpentine lineage. While the serpentine diploids appear to be unable to escape their refugia (probably because of their weak competitive abilities; [79]), the serpentine genotypes seem to have conquered surrounding non-serpentine areas at the tetraploid level (note the significant representation of the ‘serpentine’ clusters K5, K6, and K7 in adjacent non-serpentine populations; Fig. 2). The better competitive ability and higher phenotypic plasticity of the polyploids might have influenced this spread ([21], [80], see [81] for a review). Indeed, wider ecological niches of tetraploids and their ability to survive in less stable human-influenced habitats have been repeatedly documented for the genus *Knautia* [19], [82]. The spread of serpentine tetraploid genotypes far beyond serpentine areas could have been enhanced by hybridization with their non-serpentine counterparts (both lineages likely met and hybridized after human-induced deforestation). Strong introgression at the tetraploid level (marked admixture of AFLP groups in tetraploids; Figure S3) seems to be ubiquitous in the genus *Knautia* [28], [33], [35] and has also been suggested for the Slavkovský les Mts. on the basis of morphology (e.g., non-serpentine tetraploids with ‘serpentine-characteristic’ reddish corolla colour; [34]). Similar to Californian oaks [15], such ‘across-serpentine-border’ hybridization might have played a crucial role in creating new genotypes capable of colonizing new sites.

Collectively, the intricate evolutionary history of the *K. arvensis* agg. (Fig. 5) seems to be comparable only with the ‘multi-step’ evolutionary scenario of the Californian serpentine herb *Streptanthus glandulosus* (Brassicaceae), which underwent habitat restriction, area fragmentation, and subsequent independent evolution in isolated serpentine populations [12], [14], [83]. Nevertheless, the pronounced role of polyploidy in the whole evolutionary story, both as a background source of differentiation (i.e., concerted edaphic and polyploid speciation) and as a directly acting evolutionary force (i.e., independent genome duplication of serpentine relicts), seems to be a unique evolutionary pathway, firstly documented in the *K. arvensis* agg.

### Conclusions

Multifaceted interactions among ecological differentiation and polyploid evolution resulted in a unique evolutionary pattern exemplified by *Knautia arvensis* agg. A wide variety of processes and mechanisms likely took part in the rapid evolution of this complex, including isolation in Holocene refugia, repeated colonization by distinct lineages, hybridization, and recurrent polyploidization. The key role of the serpentine substrate in this scenario arises from its ability to serve as a refugium for particular lineages (in this case, relict diploid lineages). Such lineages could further evolve into distinct types, not only at the homoploid level, but also via independent genome duplication. The recurrently formed polyploids seem to be able to escape from their original refugia, indicating that the serpentine relicts are not evolutionary dead-ends but still have the potential to shape the surrounding populations. Generally, the *K. arvensis* agg. provides a unique system that illustrates the various ways in which the polyploid and serpentine evolution could act together in generating plant diversity. In addition, the genetic data strongly support previous hypotheses regarding the presence of both primary and secondary ploidy contact zones for *K. arvensis* agg., which offers exciting possibilities for addressing general questions about patterns, mechanisms, and dynamics of polyploid evolution.

## Supporting Information

Figure S1 **Second derivative of the inter cluster inertia of each number of groups (K) as estimated by the nonhierarchial K-means clustering.**
(PDF)

Figure S2 **Summary of structure 2.2 analyses based on AFLP multilocus phenotypes of 360 plants of** ***Knutia arvensis*** **agg.** Values of ln probability of the data for each number of groups (K) plotted against the K-values and Delta K values).
(PDF)

Figure S3 **Cluster membership of individuals estimated by STRUCTURE 2.2.** A – analysis of the complete dataset. B – separate STRUCTURE analysis for the relict diploid + tetraploid subgroup (grey in the plot A) resulting in six groups. Population numbers below each plot correspond to Table 1.
(PDF)

Figure S4 **Geographical location of 40 analyzed populations of** ***Knautia arvensis*** **agg. in central Europe and their phylogeographical grouping according to the structure analysis of AFLP phenotypes.**
(PDF)

Figure S5 **Principal coordinate analysis (PCoA) based on Jaccard similarity among AFLP multilocus phenotypes of** ***Knautia arvensis*** **agg. individuals.** The different colours represent the groups identified by the structure analysis (same as in Fig. S4).
(PDF)

Table S1 **Contingency table comparing the pattern in AFLP data (results of the nonhierarchical K-means clustering; clusters K1–K7) and the distribution of chloroplast haplotypes (A-M); numbers of individuals are presented in each field.**
(PDF)

Table S2 **Primary matrix of the scored AFLP fragments.**
(XLS)
