# Supplementary material for: Bringing Together Evolution on Serpentine and Polyploidy: Spatiotemporal History of the Diploid-Tetraploid Complex of Knautia arvensis (Dipsacaceae)
Source: PLoS One. 2012 Jul 5;7(7):e39988. doi: 10.1371/journal.pone.0039988 (PMC3390331; doi:10.1371/journal.pone.0039988)
Supplement: Figure S1 — Second derivative of the inter cluster inertia of each number of groups (K) as estimated by the nonhierarchial K-means clustering. (PDF) [file pone.0039988.s001.pdf]

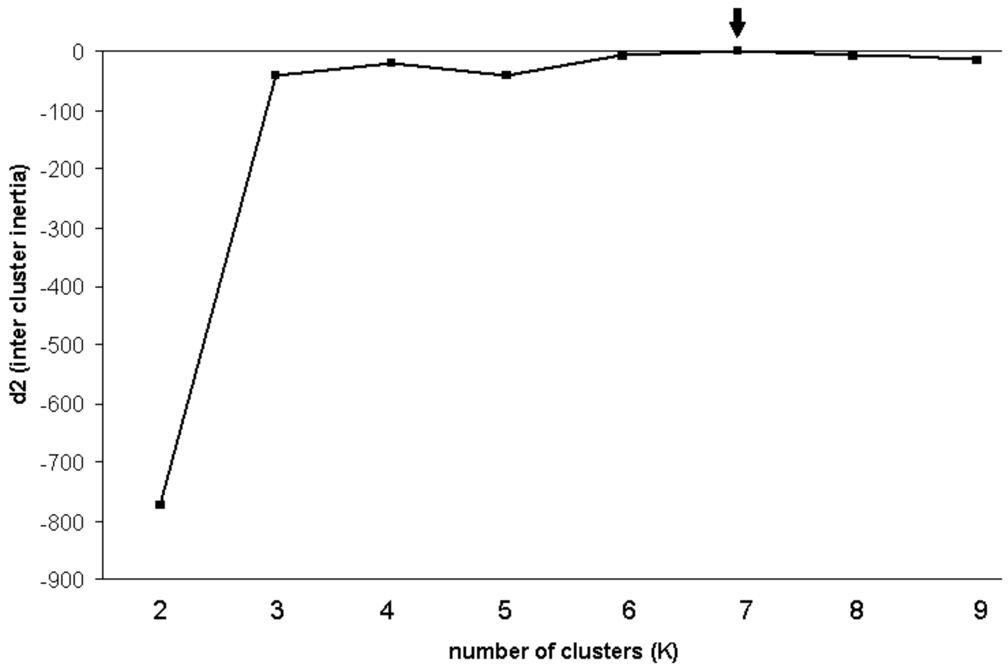

Fig. S1 Second derivative of the inter cluster inertia of each number of groups (K) as estimated by the nonhierarchial K-means clustering.
