# Supplementary material for: Bringing Together Evolution on Serpentine and Polyploidy: Spatiotemporal History of the Diploid-Tetraploid Complex of Knautia arvensis (Dipsacaceae)
Source: PLoS One. 2012 Jul 5;7(7):e39988. doi: 10.1371/journal.pone.0039988 (PMC3390331; doi:10.1371/journal.pone.0039988)
Supplement: Figure S2 — Summary of structure 2.2 analyses based on AFLP multilocus phenotypes of 360 plants of Knutia arvensis agg. Values of ln probability of the data for each number of groups (K) plotted against the K-values and Delta K values). (PDF) [file pone.0039988.s002.pdf]

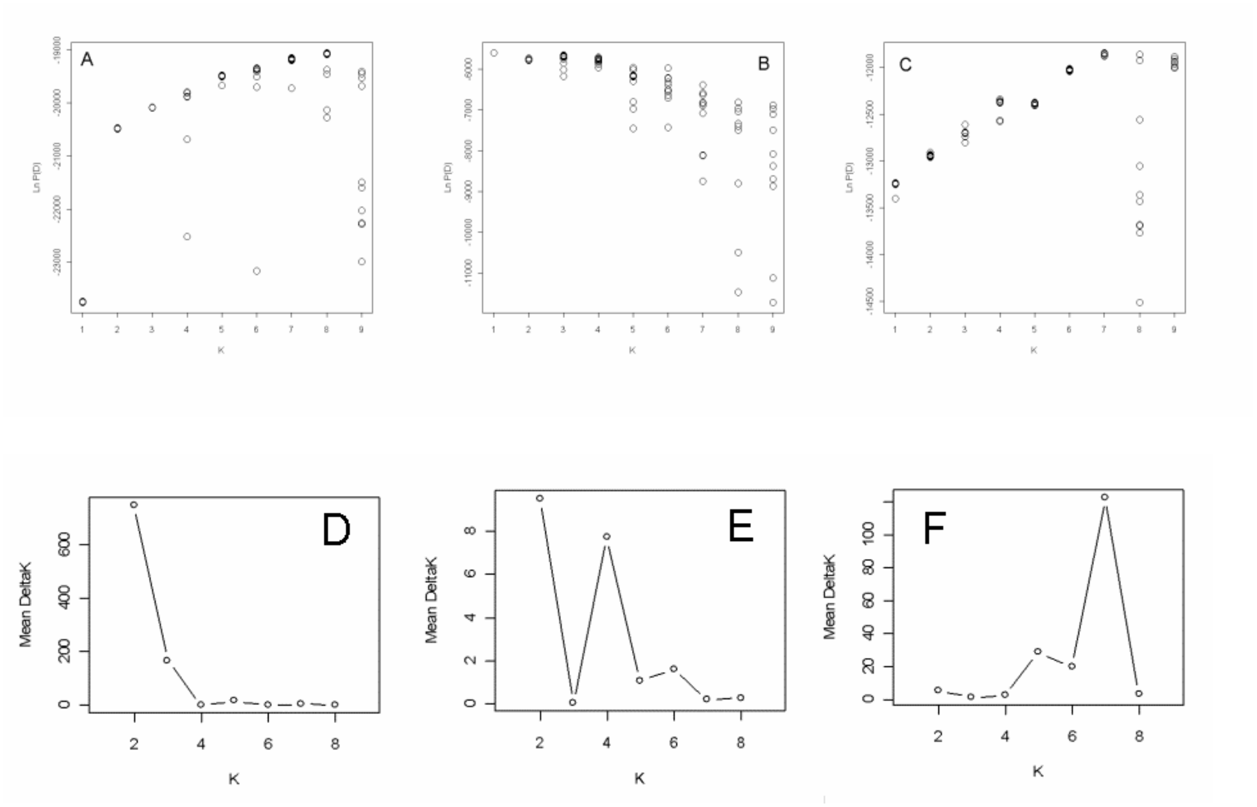

Fig. S2 Summary of STRUCTURE 2.2 analyses based on AFLP multilocus phenotypes of 360 plants of *Knutia arvensis* agg. Values of ln probability of the data for each number of groups (K) is plotted against the K-values for the whole dataset (A), and separate analyses of the non-relict diploid subgroup (B), and the relict diploid + tetraploid subgroup (C). Delta K values calculated according to Evanno et al. [55] are presented for the analysis of the whole dataset (D), and separate analyses of the non-relict diploid subgroup (E), and the relict diploid + tetraploid subgroup (F).
