# Supplementary material for: Bringing Together Evolution on Serpentine and Polyploidy: Spatiotemporal History of the Diploid-Tetraploid Complex of Knautia arvensis (Dipsacaceae)
Source: PLoS One. 2012 Jul 5;7(7):e39988. doi: 10.1371/journal.pone.0039988 (PMC3390331; doi:10.1371/journal.pone.0039988)
Supplement: Figure S3 — Cluster membership of individuals estimated by STRUCTURE 2.2. A – analysis of the complete dataset. B – separate STRUCTURE analysis for the relict diploid + tetraploid subgroup (grey in the plot A) resulting in six groups. Population numbers below each plot correspond to Table 1. (PDF) [file pone.0039988.s003.pdf]

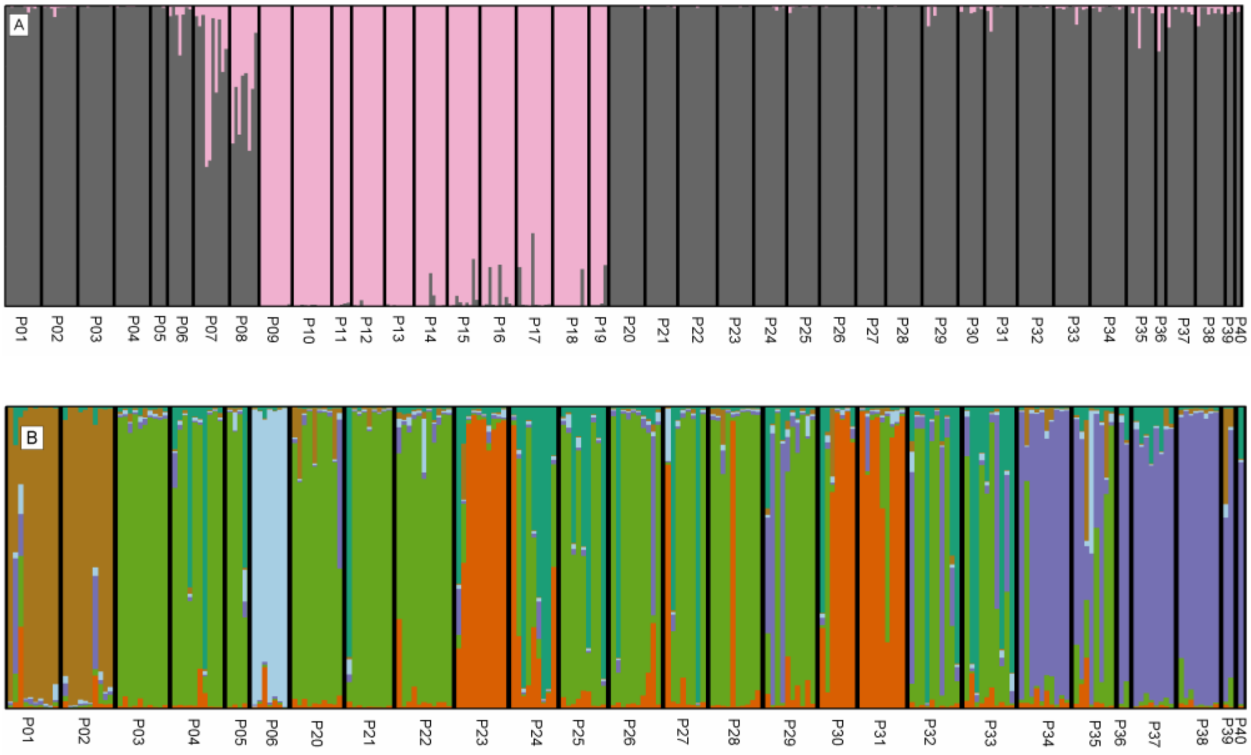

Fig. S2 Cluster membership of individuals estimated by STRUCTURE 2.2. A – analysis of the complete dataset. B – separate STRUCTURE analysis for the relict diploid + tetraploid subgroup (grey in the plot A) resulting in six groups. Population numbers below each plot correspond to Table 1.
