# Supplementary material for: Bringing Together Evolution on Serpentine and Polyploidy: Spatiotemporal History of the Diploid-Tetraploid Complex of Knautia arvensis (Dipsacaceae)
Source: PLoS One. 2012 Jul 5;7(7):e39988. doi: 10.1371/journal.pone.0039988 (PMC3390331; doi:10.1371/journal.pone.0039988)
Supplement: Figure S4 — Geographical location of 40 analyzed populations of Knautia arvensis agg. in central Europe and their phylogeographical grouping according to the structure analysis of AFLP phenotypes. (PDF) [file pone.0039988.s004.pdf]

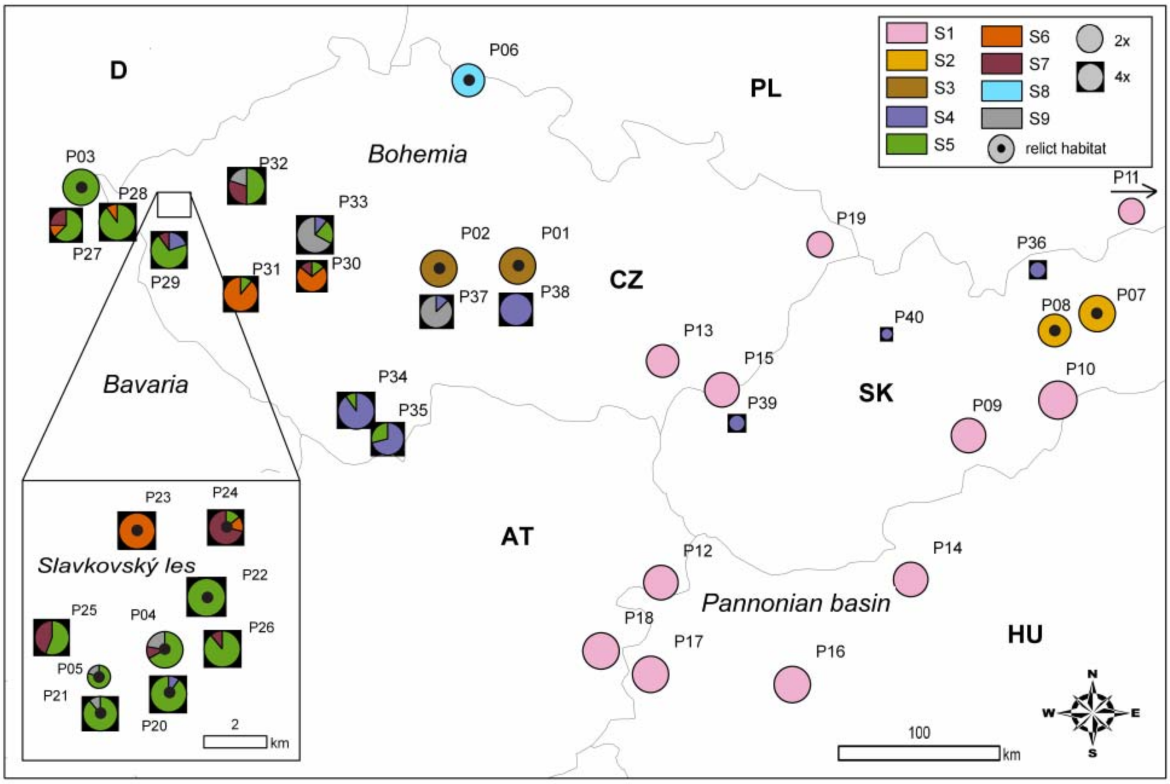

Fig. S4 Geographical location of 40 analyzed populations of *Knautia arvensis* agg. in central Europe and their phylogeographical grouping according to the STRUCTURE analysis of AFLP phenotypes. Pie charts represent the proportion of individuals belonging to each of the nine detected groups (S1-S9). The size of the pie chart reflects sample size. The inset displays the situation in the Slavkovský les serpentine area.
