# Supplementary material for: Bringing Together Evolution on Serpentine and Polyploidy: Spatiotemporal History of the Diploid-Tetraploid Complex of Knautia arvensis (Dipsacaceae)
Source: PLoS One. 2012 Jul 5;7(7):e39988. doi: 10.1371/journal.pone.0039988 (PMC3390331; doi:10.1371/journal.pone.0039988)
Supplement: Figure S5 — Principal coordinate analysis (PCoA) based on Jaccard similarity among AFLP multilocus phenotypes of Knautia arvensis agg. individuals. The different colours represent the groups identified by the structure analysis (same as in Fig. S4). (PDF) [file pone.0039988.s005.pdf]

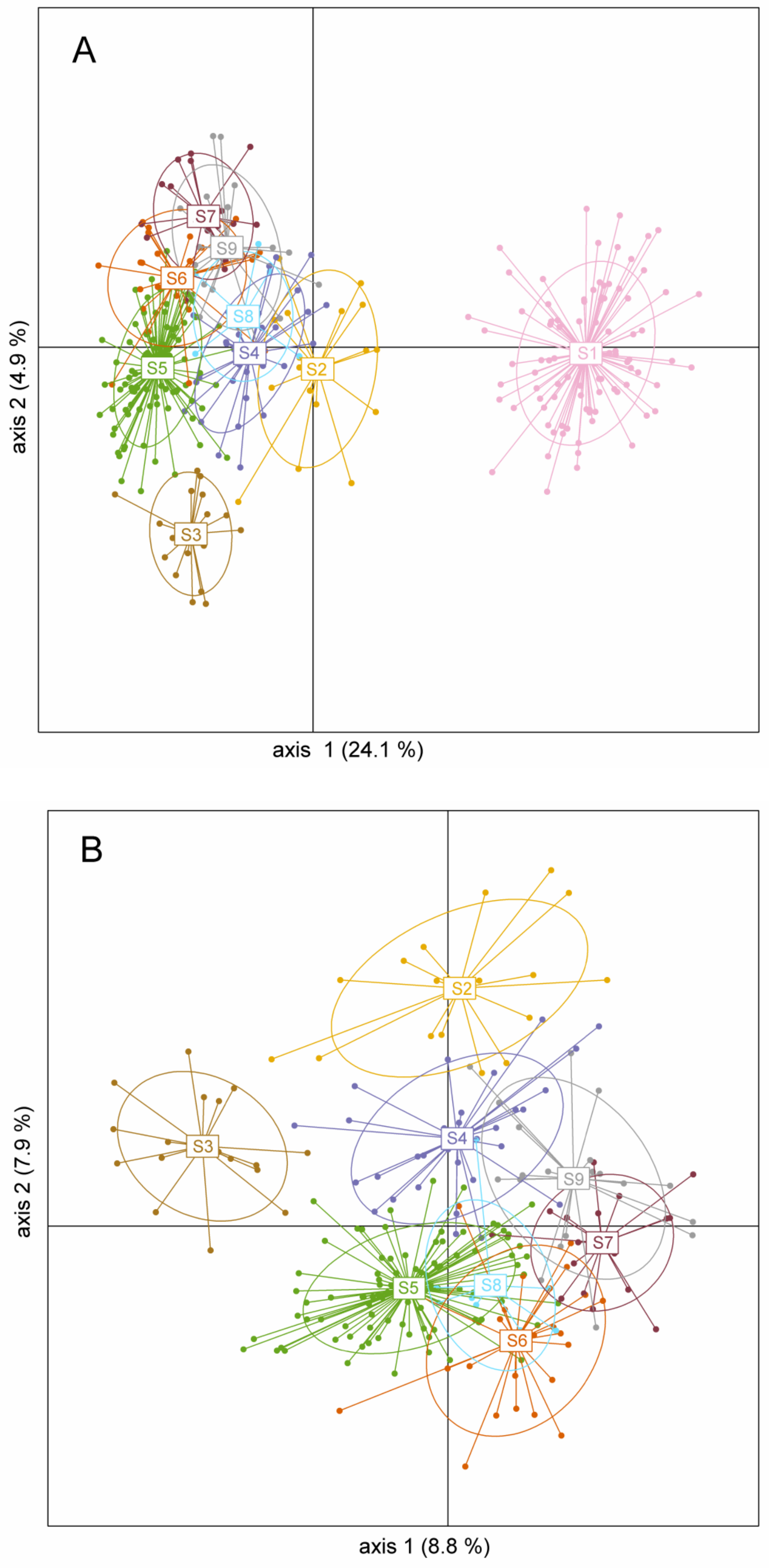
A
S7
S9
S6
S8
S4
S5
S2
S1
S3
axis 2 (4.9 %)
axis 1 (24.1 %)
B
S2
S3
S4
S9
S7
S5
S8
S6
axis 2 (7.9 %)
axis 1 (8.8 %)

Fig. S5 Principal coordinate analysis (PCoA) based on Jaccard similarity among AFLP multilocus phenotypes of *Knautia arvensis* agg. individuals. Plot A displays the arrangement along the first and second axis in the whole dataset while the plot B displays the arrangement after the omission of the most divergent group S1 (i.e., the non-relict diploids). The different colours represent the groups identified by the STRUCTURE analysis (same as in Fig. S4). The centroid of each group and its connection with other points are displayed as well as an ellipse reflecting the variance of the group and the covariance on the axes.
