# Supplementary material for: Bringing Together Evolution on Serpentine and Polyploidy: Spatiotemporal History of the Diploid-Tetraploid Complex of Knautia arvensis (Dipsacaceae)
Source: PLoS One. 2012 Jul 5;7(7):e39988. doi: 10.1371/journal.pone.0039988 (PMC3390331; doi:10.1371/journal.pone.0039988)
Supplement: Table S1 — Contingency table comparing the pattern in AFLP data (results of the nonhierarchical K-means clustering; clusters K1–K7) and the distribution of chloroplast haplotypes (A-M); numbers of individuals are presented in each field. (PDF) [file pone.0039988.s006.pdf]

Table S1 Contingency table comparing the pattern in AFLP data (results of the nonhierarchical K-means clustering; clusters K1-K7) and the distribution of chloroplast haplotypes (A-M); numbers of individuals are presented in each field.

	A	B	C	D	E	F	G	H	I	J	K	L	M
K1	7									1			1
K2	1												
K3	1					3		2					
K4	6	3	1			3							
K5	10	3		2							2		
K6	7	1		1	4	1		2					
K7	6	1		2		1	1	1	2			1	
